# Assessment of Renal Changes in Bardet–Biedl Syndrome with Particular Emphasis on Kidney Size, Function, and Blood Pressure Control

**DOI:** 10.34763/jmotherandchild.20263001.d-26-00009

**Published:** 2026-06-28

**Authors:** Zofia Moskal, Agnieszka Szmigielska

**Affiliations:** Extracurricular medical student scientific association, Department of Pediatrics and Nephrology, Medical University of Warsaw, Warsaw, Poland; Department of Pediatrics and Nephrology, Medical University of Warsaw, Warsaw, Poland

**Keywords:** Bardet-Biedl syndrome, dysplastic kidney, hypertension, obesity, ciliopathy

## Abstract

**Introduction:**

Bardet-Biedl syndrome (BBS) is a genetically heterogeneous ciliopathy characterized by obesity, retinal dystrophy, cognitive impairment, hypogonadism, and congenital renal anomalies, which represent a leading cause of morbidity and mortality in affected patients.

**Material and methods:**

We followed four patients with BBS (three boys, one girl) from birth to ten years of age. In all children, we assessed physical development, laboratory tests, including assessment of renal function, renal ultrasound examination, and blood pressure control.

**Results:**

Patients (three males) with Bardet-Biedl syndrome, demonstrated prenatal and postnatal evidence of structural and functional renal abnormalities, including kidney injury and bilateral renal dysplasia. Neonatal serum creatinine ranged from 0.70 to 1.66 mg/dL, with persistently abnormal renal morphology on follow-up imaging and variable but overall stable renal function (latest creatinine 0.68–1.03 mg/dL). Arterial hypertension occurred in one male and one female patient and was effectively controlled with angiotensin-converting enzyme inhibitor therapy. All patients exhibited multisystem involvement characteristic of ciliopathy, including polydactyly, obesity, ophthalmologic abnormalities, neurodevelopmental impairment, and hypogonadism in three of four individuals.

**Conclusions:**

The broad spectrum of renal manifestations in BBS underscoring the importance of early diagnosis and individualized nephrological monitoring.

## Introduction

Bardet-Biedl syndrome (BBS) is a rare autosomal recessive primary ciliopathy characterized by multisystem involvement, with an estimated prevalence of approximately one in 125,000–175,000 live births in European populations [1]. Ciliopathies are disorders caused by dysfunctional primary (non-motile) cilia, organelles critical for cellular signal transduction and tissue development. Defects in motile cilia, by contrast, are associated with conditions such as primary ciliary dyskinesia, which predominantly affects respiratory function and left–right body patterning [2]. BBS exhibits genetic heterogeneity, with pathogenic variants identified in at least 26–27 genes to date. Most encode components of the BBSome or associated chaperonin complex, which are required for ciliary trafficking and BBSome assembly [3]. Mutations in *BBS1* and *BBS10* are the most frequently identified in many populations, although allele frequencies vary ethnically [4]. Diagnosis is clinical and typically requires the presence of four major features or three major plus two minor features [3]. Major features include retinal dystrophy, central obesity, postaxial polydactyly, cognitive impairment, genital anomalies, and renal anomalies [3, 4]. Secondary features include developmental delay, speech abnormalities, diabetes mellitus, dental anomalies, ataxia/poor coordination, congenital heart disease, and anosmia/hyposmia [3]. Retinal dystrophy is among the most penetrant features, with rod–cone degeneration typically presenting in childhood as nyctalopia and progressive visual loss, often resulting in legal blindness by early adulthood in many patients [5, 6, 7]. Truncal obesity develops early in life despite normal birth weight and is present in the majority of affected individuals, contributing to metabolic comorbidities such as insulin resistance and type 2 diabetes mellitus [6]. Postaxial polydactyly is a common congenital anomaly and may be evident at birth [4, 8, 9]. Cognitive impairment and developmental delay are frequently observed, often in the mild to moderate range [4, 10, 13]. Hypogonadism, particularly in males, is common and reflects the role of ciliary signalling in genitourinary development [4, 11, 12, 14]. Renal involvement is highly variable, ranging from structural anomalies (e.g., dysplasia, cystic changes) to functional impairment, and is a leading contributor to morbidity and mortality in BBS [3]. Regular multidisciplinary follow-up, including ophthalmologic and nephrological monitoring, is essential given the progressive nature and interindividual variability of manifestations [5, 12].

The aim of the study was to assess renal changes in Bardet-Biedl syndrome with particular emphasis on kidney size, function, and blood pressure control.

## Material and methods

The study group comprised four children (three boys, one girl) diagnosed with Bardet-Biedl syndrome, who were admitted to a Tertiary Paediatric Nephrology Department for comprehensive evaluation of renal abnormalities. The diagnosis of BBS was established based on characteristic clinical features and confirmed by genetic testing in three patients. All patients had abnormal renal findings detected on prenatal ultrasonography and persistent impairment of renal function after birth.

### Clinical and Laboratory Assessment

All children underwent detailed clinical evaluation, including assessment of growth parameters, blood pressure measurements performed according to age- and height-adjusted paediatric standards. Arterial hypertension was diagnosed based on repeated measurements exceeding the 95th percentile for age, sex, and height. Information on the onset of hypertension and the need for antihypertensive therapy, particularly angiotensin-converting enzyme (ACE) inhibitors, was recorded. Laboratory investigations included serial measurements of serum creatinine and urea from the neonatal period through long-term follow-up.

### Imaging Studies

All patients underwent prenatal and postnatal renal ultrasonography. Postnatal ultrasound examinations were performed at regular intervals and included assessment of renal size, parenchymal echogenicity, corticomedullary differentiation, and the presence of cystic lesions or other structural abnormalities. Kidney length was compared with age-related normative values to classify kidneys as small (hypoplastic/dysplastic), normal in size, or enlarged.

### Follow-up and Outcome Measures

Patients were followed longitudinally for up to ten years. The primary outcome measures were changes in renal function (serum creatinine, urea) and progression of CKD. Secondary outcome measures included the development of arterial hypertension and its treatment, as well as the evolution of renal size and structure on serial ultrasonography.

## Results

Four term-born patients (three males and one female) with Bardet-Biedl syndrome (BBS) spectrum disorder were evaluated. Gestational age ranged from 40 to 41 weeks, and birth weight varied between 3010 g and 4100 g. Genetic analysis was performed using a targeted **next-generation sequencing (NGS)** panel comprising 23 genes associated with Bardet-Biedl syndrome. All pathogenic and likely pathogenic variants identified by panel NGS sequencing were validated using Sanger sequencing, which served as a confirmatory method. Genetic results in three patients (human reference genome version GRCh38):

Patient 1: Compound heterozygous pathogenic variants were identified in the *MKKS* gene:

MKKSNM_170784.3:c.[595_596del];[1436C>G];NP_740754.1:p.[(Ser199PhefsTer22)];[(Ser479Ter)].

Patient 2: The same compound heterozygous variants in the *MKKS* gene were detected as in patient 1:

MKKSNM_170784.3:c.[595_596del];[1436C>G];NP_740754.1:p.[(Ser199PhefsTer22)];[(Ser479Ter)].

Patient 3: Compound heterozygous variants were identified in the *BBS1* gene:

BBS1NM_024649.5:c.[268_272dup];[479+2T>G]NP_078925.3:p.[(Phe92ProfsTer61)];[?]

In the fourth patient, the diagnosis of BBS was made based on clinical symptoms; genetic testing is not available.

Prenatal ultrasonography revealed renal abnormalities in all patients, although with heterogeneous phenotypic expression. Findings included bilateral renal enlargement with cystic changes, small dysplastic kidneys, increased parenchymal echogenicity, and suspected polycystic morphology. Postnatal imaging confirmed structural renal dysplasia in all cases. Three patients demonstrated bilaterally small dysplastic kidneys, while one male patient exhibited enlarged dysplastic kidneys with multiple cysts and absent corticomedullary differentiation (CMD). Loss of CMD was documented in three patients, indicating impaired nephron maturation and structural disorganization. Peripheral cortical cysts were observed in two individuals. At latest follow-up, three patients exhibited persistent bilateral renal hypodysplasia with reduced renal size, whereas one male patient continued to demonstrate nephromegaly with cystic remodelling and absent CMD, suggesting phenotypic variability within the cohort.

Neonatal renal function was variably impaired. Serum creatinine at birth ranged from 0.70 to 1.66 mg/dL, with the highest value observed in a male neonate presenting with enlarged cystic kidneys. Urea concentrations at birth ranged from 39 to 55 mg/dL. At most recent evaluation, serum creatinine ranged between 0.68 and 1.03 mg/dL, while urea levels ranged from 24.7 to 46.4 mg/dL. Two patients demonstrated persistently higher nitrogen retention parameters, consistent with chronic kidney involvement. Overall, renal function appeared relatively stable during follow-up, although structural abnormalities persisted in all patients.

Arterial hypertension was documented in one male and one female patient. Both individuals were treated with angiotensin-converting enzyme (ACE) inhibitors, resulting in satisfactory blood pressure control and stabilization of renal function parameters during follow-up.

Polydactyly and early-onset obesity were present in all patients, consistent with the classical BBS phenotype. Ophthalmologic involvement was universal, manifesting as rod–cone dystrophy in two patients, nyctalopia in one female patient, and retinal pigmentary abnormalities in one male patient. Neurodevelopmental impairment was observed in all individuals, including cognitive impairment in three patients and global developmental delay in one. Hypogonadism was documented in three patients, whereas one female patient did not exhibit endocrine or genital abnormalities.

The research results are presented in Table 1.

**Table 1.** Clinical, Genetic, Imaging, and Laboratory Characteristics of Four Paediatric Patients with Bardet-Biedl Syndrome.

**Parameter**	**Case 1**	**Case 2**	**Case 3**	**Case 4**
Sex	Male	Male	Female	Male
Gestational age Birth weight	40 wks 3660 g	40 wks 4100 g	41 wks 3010 g	41 wks 4060 g
Genetic findings	MKKS	MKKS	BBS1	*
Prenatal renal US	Enlarged kidneys with cysts	Small dysplastic kidneys	Increased echogenicity of kidneys	Polycystic kidneys suspected
Postnatal renal US	Enlarged dysplastic kidneys, absent CMD, cysts	Small dysplastic kidneys	Normal size, absent CMD, small cysts	Small dysplastic kidneys, multiple peripheral cysts, absent CMD
Latest renal US	Enlarged kidneys, absent CMD, cysts	Small dysplastic kidneys	Small dysplastic kidneys	Small dysplastic kidneys
Serum creatinine at birth (mg/dl)	1.66	0.70	1.04	1.25
Urea at birth (mg/dl)	55	39	42	51
Latest serum creatinine (mg/dl)	0.85	1.03	0.68	0,89
Latest urea (mg/dl)	24.7	46.4	26.7	42
Polydactyly, obesity	Yes	Yes	Yes	Yes
Hypertension	Yes	No	Yes	No
Ophthalmological findings	Rod-cone dystrophy	Rod-cone dystrophy	Nyctalopia	Retinal pigment abnormalities
Neurodevelopment	Cognitive impairment	Cognitive impairment	Cognitive impairment	Global developmental delay
Endocrine / genital anomalies	Hypogonadism	Hypogonadism	Not yet	Hypogonadism

Abbreviations: BBS – Bardet-Biedl syndrome; CMD – cortico-medullary differentiation; CKD – chronic kidney disease; US – ultrasonography.

* Genetic testing is not available.

## Discussion

In children with Bardet-Biedl syndrome (BBS), the development and progression of chronic kidney disease (CKD) are influenced by both congenital renal abnormalities and acquired risk factors such as arterial hypertension and obesity [15]. Renal involvement is a cardinal feature of BBS and represents one of the main determinants of long-term morbidity and mortality. Structural or functional kidney abnormalities are reported in approximately 50–80% of patients, and 25–40% develop CKD during childhood or adulthood, with a smaller proportion progressing to end-stage kidney disease [16, 17]. The postnatal renal phenotype and the presence of arterial hypertension appear to play a pivotal role in CKD progression. In full-term neonates, normal renal length is approximately 50 mm, and kidneys should demonstrate preserved corticomedullary differentiation and normal echogenicity. In BBS, however, renal size and morphology are highly variable. Prenatal and postnatal ultrasonography may reveal small (hypoplastic or dysplastic) kidneys, enlarged kidneys with abnormal echostructure, or kidneys of normal size containing single or multiple cysts; in some cases, the cystic burden raises suspicion of polycystic kidney disease [16, 17]. Loss of corticomedullary differentiation, increased echogenicity, persistent fetal lobulation, and medullary or cortical cysts are frequently observed and reflect disturbed nephrogenesis related to the underlying ciliopathy [17, 18]. Small, dysplastic kidneys are presumed to contain a reduced number of nephrons, which predisposes to glomerular hyperfiltration, early development of arterial hypertension, and more rapid progression of CKD. Conversely, enlarged kidneys with abnormal parenchymal structure may also generate hypertension and progressive renal dysfunction; however, the rate of CKD progression in such cases may be slower, likely due to a relatively greater nephron mass. Thus, both reduced and increased renal size in BBS, particularly when accompanied by abnormal echostructure, are clinically significant and require long-term nephrological follow-up. In the described cohort of children with BBS, abnormal renal morphology was already detected on prenatal ultrasonography [19, 20]. The kidneys were reported as enlarged, small, or of normal size with cystic lesions. Postnatal ultrasound confirmed structural abnormalities. During long-term follow-up, three of four children developed small, dysplastic kidneys, whereas in one child the kidneys remained enlarged with blurred corticomedullary differentiation [21, 22].

All patients demonstrated elevated serum urea and creatinine concentrations at birth, which gradually decreased during the neonatal period but never normalized [23]. After approximately two years of age, a secondary gradual increase in serum creatinine was observed. Over the subsequent ten years of observation, renal function parameters remained relatively stable, indicating slow progression of CKD. Laboratory evaluation did not reveal typical complications of advanced CKD, such as anaemia or disturbances of calcium–phosphate metabolism, suggesting preserved endocrine renal function at this stage.

Arterial hypertension is a common complication of BBS, reported in approximately 20–35% of paediatric patients and more than 30% of adults, with prevalence increasing with age and declining Glomerular Filtration Rate [17, 24]. In the present group, hypertension developed in two children (50%) before the age of five years and required initiation of angiotensin-converting enzyme inhibitor therapy. Hypertension acts as both a consequence of reduced nephron number and a major promoter of further renal damage, accelerating CKD progression and increasing cardiovascular risk.

Severe, early-onset obesity is a hallmark of BBS and affects more than 70–80% of patients. Obesity constitutes an independent risk factor for CKD through mechanisms including glomerular hyperfiltration, increased intraglomerular pressure, and subsequent glomerulosclerosis, and it also contributes to the development of hypertension and metabolic disturbances [25]. These factors synergistically exacerbate renal injury in individuals with an already compromised nephron endowment due to congenital renal malformations. Furthermore, intellectual disability commonly associated with BBS may impede accurate blood pressure measurement, adherence to pharmacological treatment, and compliance with dietary and lifestyle recommendations. Consequently, the progression of CKD may accelerate, particularly during adolescence, when metabolic burden and blood pressure tend to increase. Regular, lifelong surveillance of renal structure and function, together with early management of hypertension and obesity, is therefore essential in patients with BBS to delay CKD progression and reduce long-term complications.

## Conclusion

In this small cohort of children with Bardet-Biedl syndrome, the progression of chronic kidney disease appeared to be associated with renal morphology on ultrasonography, blood pressure control, and body weight status. Abnormal renal imaging findings, hypertension, pathological obesity, as well as challenges related to dietary adherence and medication management, were observed more frequently among patients with CKD. Given the limited sample size, these findings should be interpreted with caution and considered hypothesis-generating. Further studies in larger cohorts are required to better define these associations. Early recognition and management of potentially modifiable factors may be important in mitigating renal disease progression in paediatric BBS patients.
